# [^68^Ga]FSC-(RGD)_3_ a trimeric RGD peptide for imaging α_v_β_3_ integrin expression based on a novel siderophore derived chelating scaffold—synthesis and evaluation

**DOI:** 10.1016/j.nucmedbio.2014.10.001

**Published:** 2015-02

**Authors:** Peter A. Knetsch, Chuangyan Zhai, Christine Rangger, Michael Blatzer, Hubertus Haas, Piriya Kaeopookum, Roland Haubner, Clemens Decristoforo

**Affiliations:** a Department of Nuclear Medicine, Medical University Innsbruck, Innsbruck, Austria; b Division of Molecular Biology, Medical University Innsbruck, Innsbruck, Austria; c Ministry of Science and Technology (MOST), Thailand Institute of Nuclear Technology (TINT), Bangkok, Thailand

**Keywords:** 68Ga, Siderophors, Bifunctional chelator, RGD, αvβ3 integrin

## Abstract

Over the last years Gallium-68 (^68^Ga) has received tremendous attention for labeling of radiopharmaceuticals for positron emission tomography (PET). ^68^Ga labeling of biomolecules is currently based on bifunctional chelators containing aminocarboxylates (mainly DOTA and NOTA). We have recently shown that cyclic peptide siderophores have very good complexing properties for ^68^Ga resulting in high specific activities and excellent metabolic stabilities, in particular triacetylfusarinine-C (TAFC). We postulated, that, starting from its deacetylated form (Fusarinine-C (FSC)) trimeric bioconjugates are directly accessible to develop novel targeting peptide based ^68^Ga labeled radiopharmaceuticals. As proof of principle we report on the synthesis and ^68^Ga-radiolabeling of a trimeric FSC-RGD conjugate, [^68^Ga]FSC-(RGD)_3_, targeting α_v_β_3_ integrin, which is highly expressed during tumor-induced angiogenesis.

Synthesis of the RGD peptide was carried out applying solid phase peptide synthesis (SPPS), followed by the coupling to the siderophore [Fe]FSC via *in situ* activation using HATU/HOAt and DIPEA. Subsequent demetalation allowed radiolabeling of FSC-(RGD)_3_ with ^68^Ga. The radiolabeling procedure was optimized regarding peptide amount, reaction time, temperature as well buffer systems. For *in vitro* evaluation partition coefficient, protein binding, serum stability, α_v_β_3_ integrin binding affinity, and tumor cell uptake were determined. For *in vitro* tests as well as for the biodistribution studies α_v_β_3_ positive human melanoma M21 and α_v_β_3_ negative M21-L cells were used.

[^68^Ga]FSC-(RGD)_3_ was prepared with high radiochemical yield (> 98%). Distribution coefficient was − 3.6 revealing a hydrophilic character, and an IC_50_ value of 1.8 ± 0.6 nM was determined indicating a high binding affinity for α_v_β_3_ integrin. [^68^Ga]FSC-(RGD)_3_ was stable in PBS (pH 7.4), FeCl_3_- and DTPA-solution as well as in fresh human serum at 37 °C for 2 hours. Biodistribution assay confirmed the receptor specific uptake found *in vitro*. Uptake in the α_v_β_3_ positive tumor was 4.3% ID/g 60 min p.i. which was 3-fold higher than the monomeric [^68^Ga]NODAGA-RGD. Tumor to blood ratio of approx. 8 and tumor to muscle ratio of approx. 7 were observed. [^68^Ga]FSC-(RGD)_3_ serves as an example for the feasibility of a novel class of bifunctional chelators based on cyclic peptide siderophores and shows excellent targeting properties for α_v_β_3_ integrin *in vivo* for imaging tumor-induced neovascularization.

## 1 Introduction

With the increasing availability of ^68^Ge/^68^Ga generators, ^68^Ga-labeled radiopharmaceuticals, especially based on peptides, are coming into the focus of research interest in the field of radiopharmaceutical sciences [1]. The advantages lie in the easy access of a PET radionuclide without the need of an in-house cyclotron and the straightforward labeling strategies which are optimal for synthesis automation. The most widely applied chelating systems for a variety of radiometals, including ^68^Ga, is 1,4,7,10-tetraazacyclododecane-1,4,7,10-tetraacetic acid (DOTA). Whereas for somatostatin analogues ^68^Ga-labeled DOTA-conjugates revealed superior properties regarding pharmacokinetics and receptor affinity in comparison to other radiometals such as ^111^In [2], for other peptides imaging properties of the ^68^Ga labeled construct were inferior to other radiometals.(e.g. [3]).

Even though the field of ^68^Ga radiopharmaceuticals is rapidly expanding [4], the number of alternatives to DOTA for conjugation to biomolecules and subsequent ^68^Ga-labeling is still limited, and novel bifunctional chelators could help to further advance the development of ^68^Ga-based radiopharmaceuticals.

**Fig. 1:**
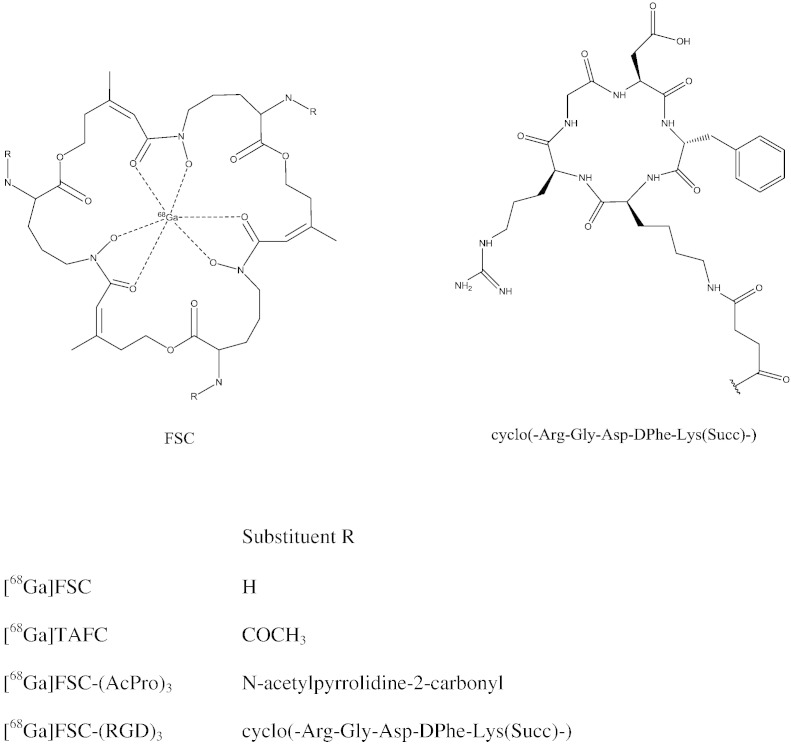
Proposed structures of [^68^Ga]FSC, [^68^Ga]TAFC, [^68^Ga]FSC-(Ac-Pro)_3_ and [^68^Ga]FSC-(RGD)_3_.

Siderophores are low molecular-mass chelators with very high affinity for ferric ions which are utilized by bacteria, fungi, and plants for iron acquisition and storage [5]. Recently we demonstrated that ^68^Ga-labeled peptide siderophores, in particular triacetylfusarinine-C (TAFC), are potential agents for early imaging of invasive pulmonary aspergillosis (IPA) due to their high specific uptake in *Aspergillus fumigatus* (*A*.*f*.) [6], [7]. TAFC, a representative of the class of hydroxamate siderophores, binds ^68^Ga with high affinity at room temperature (RT) within minutes achieving very high specific activities. It is composed of three *N^5^*-hydroxy-L-ornithine and three anhydromevalonic acid groups (for structure see Fig. 1) forming hexacoordinate Fe^3 +^ and Ga^3 +^ complexes in a tris-bidentate form involving the three hydroxamate functions of the molecule, respectively.

We were interested whether such cyclic siderophores could be used as a basis for preparing targeted bioconjugates for ^68^Ga labeling. The siderophore fusarinine-C (FSC) is the deacetylated form of TAFC. We postulated that the three primary amines of FSC could be used for conjugation to biomolecules such as RGD peptides. Whereas [^68^Ga]TAFC was extremely stable in serum showing no sign of degradation and even no release of ^68^Ga when challenged with FeCl_3_- or DTPA-solution, [^68^Ga]FSC released up to 20% of complexed activity [8]. Based on these data it was conceivable that acylation as well as alkylation of the amines of FSC may have a stabilizing effect on the siderophore and could be used for conjugation of targeting sequences.

To prove this hypothesis, we initially attempted to prepare a simple trimeric conjugate using acetyl prolin to test various strategies for conjugation of peptidic structures to FSC. This was followed by conjugation of an RGD peptide as a well established targeting sequence. A great variety of different radiolabeled RGD-peptides have been described labeled with halogens as well as with various radiometals (for review see [9], [10]) and therefore cyclic RGD peptides can serve as an excellent model for targeting applications. They bind the α_v_β_3_ integrin found on activated endothelial cells during tumor-induced angiogenesis and also being highly expressed on various tumor types, e.g. melanoma, glioblastoma, pancreatic-, and cervical cancer [11]. In this study we report on the feasibility of preparing a trimeric FSC-RGD conjugate for ^68^Ga labeling and the initial comparison of its biological properties with the established monomeric [^68^Ga]NODAGA-RGD [12].

## 2 Materials and methods

### 2.1 General

All chemicals were used as supplied without further purification. 9-Fluorenylmethoxycarbonyl (Fmoc) protected amino acids were purchased from Novabiochem (La Jolla, CA, USA). The solid support tritylchloride polystyrene resin (TCP resin) was obtained from PepChem (Reutlingen, Germany). Coupling reagents 1-Hydroxy-7-azabenzotriazole (HOAt) and *O*-(7-azabenzotriazol-1-yl)-1,1,3,3-tetramethyl uroniumhexafluorophosphate (HATU) were purchased from GenScript Corporation (Piscataway, NJ, USA). All other chemicals were obtained from VWR International GmbH (Vienna, Austria) or Sigma-Aldrich Handels GmbH (Vienna, Austria). Human M21 and M21-L melanoma cells were a kind gift from D. A. Cheresh, Departments of Immunology and Vascular Biology, The Scripps Research Institute, La Jolla, CA, USA.

Reversed-phase high performance liquid chromatography (RP-HPLC) analysis was performed with an UltiMate 3000 RS HPLC pump, an UltiMate 3000 RS Column compartment (column oven temperature was set at 25 °C), an UltiMate 3000 Variable Wavelength detector (Dionex, Gemering, Germany), and a Bioscan radiometric detector (Bioscan, Washington DC, USA). A Vydac 218 TP5215, 150 × 3.0 mm column (SRD, Vienna, Austria), flow rates of 1.0 ml/min, and UV detection at 220 nm were employed with the following acetonitrile (CH_3_CN)/H_2_O/0.1% TFA gradients: 0–2.0 min 0% CH_3_CN, 2.0–16.0 min 0–40% CH_3_CN (gradient A); 0–2.0 min 30% CH_3_CN, 2.0–18.0 min 30–80% CH_3_CN (gradient B); 0–2.0 min 0% CH_3_CN, 2.0–18.0 min 0–80% CH_3_CN (gradient C); and 0–0.5 min 0% CH_3_CN, 0.5–7.0 min 0–55% CH_3_CN (gradient D).

Cyclo(-Arg(Pbf)-Gly-Asp(*O*tBu)-dPhe-Lys(Succ)-) was isolated via preparative RP-HPLC using a Gilson 322 HPLC pump with Gilson UV/VIS-155 detector (Gilson International B.V., Limburg, Germany) and a MultoHigh 100 RP 18 5 μm, 250 × 10 mm column (CS–Chromatographie Service GmbH, Langerwehe, Germany). Flow rate was 5.0 ml/min. The used CH_3_CN/H_2_O/0.1% TFA gradient was as follows: 0–1.0 min 30% CH_3_CN, 1.0–21.0 min 30–80% CH_3_CN (gradient E). Purification of Fusarinine-C was carried out via preparative RP-HPLC using a P580 HPLC pump (Dionex, Gemering, Germany), a Gynkotek Wavelength detector (Gynkotek, Gemering, Germany), and a Seibersdorf Nucleosil 120-10 C18, 250 × 8 mm column (Austrian Institute of Technology, Seibersdorf, Austria) with a flow rate of 5 ml/min and a gradient with following specifications: 0–1.0 min 5% CH_3_CN, 1.0–14.0 min 5–40% CH_3_CN (gradient F). [Fe]FSC-(RGD)_3_ as well as FSC-(RGD)_3_ were purified by semipreparative RP-HPLC employing the same system components as described before for the siderophore but using instead a Bischoff Nucleosil 120-5-C18 5 μm, 250 × 4.6 mm column (Bischoff Analysentechnik und -geräte GmbH, Leonberg, Germany) with a flow rate of 1.5 ml/min. The gradient was: 0–1.0 min 0% CH_3_CN, 1.0–2.0 min 0–25% CH_3_CN, 2.0–18.0 min 25–35% CH_3_CN (gradient G).

Matrix-assisted laser desorption/ionization–time of flight–mass spectrometry (MALDI-TOF-MS) was carried out using α-cyano-4-hydroxy-cinnamic acid as the matrix and a Bruker Ultraflex mass spectrometer (Ultraflex MALDI TOF-TOF; Bruker Daltonics, Bremen, Germany). All spectra were recorded by summarizing 600 laser shots, using a 337 nm nitrogen laser with a pulse of 50 Hz. For data processing Flex Analysis 2.4 software was used.

The radioactivity of *in vitro* as well as *in vivo* samples was measured using a 2480 Automatic Gamma Counter Wizard^2^ 3″ (Perkin Elmer, Vienna, Austria).

The ^68^Ga-generator was purchased from Eckert & Ziegler Strahlen- und Medizintechnik AG (Berlin, Germany) with a nominal activity of 1100 MBq and was eluted with 0.1 M HCl solution (Rotem Industries Ltd., Beer-Sheva, Israel) carrying out the fractionated elution approach [13].

### 2.2 Cyclo(-Arg(Pbf)-Gly-Asp(*O*tBu)-dPhe-Lys-)

Detailed synthesis procedure is described elsewhere [14]. Briefly, the linear RGD-peptide was synthesized on a solid support using a tritylchloride resin (TCP resin) and Fmoc protocols. Protecting groups were 2,2,4,6,7-pentamethyldihydrobenzofuran-5-sulfonyl (Pbf) for arginine, tert-butyl (tBu) for aspartic acid, and benzyloxycarbonyl (Z) for lysine. Cyclization of the peptide was performed in dimethylformamide (DMF) in the presence of diphenylphosphoryl azide (DPPA) and sodium hydrogen carbonate (NaHCO_3_) as solid base. Subsequent deprotection of the Lysine side chain was carried out under hydrogen atmosphere in the presence of an activated charcoal palladium catalyst in *N*,*N*-dimethylacetamide (DMA) resulting in cyclo(-Arg(Pbf)-Gly-Asp(*O*tBu)-dPhe-Lys-).

### 2.3 Cyclo(-Arg(Pbf)-Gly-Asp(*O*tBu)-dPhe-Lys(Succ)-)

To cyclo(-Arg(Pbf)-Gly-Asp(*O*tBu)-dPhe-Lys-) (100 mg, 0.11 mmol) dissolved in 5 ml DMF, 2 eq of succinic anhydride (22 mg, 0.22 mmol) were added. The mixture was stirred for 12 h at RT. Subsequently the solvent was reduced *in vacuo* and the peptide precipitated by addition of water. The crude product cyclo(-Arg(Pbf)-Gly-Asp(*O*tBu)-dPhe-Lys(Succ)-) was washed, dried and finally purified via preparative RP-HPLC (gradient E): t_R_ = 15.3 min, yield: 62 mg cyclo(-Arg(Pbf)-Gly-Asp(*O*tBu)-dPhe-Lys(Succ)-) (0.06 mmol, 56% of theoretical yield). RP-HPLC (gradient B): t_R_ = 7.9 min. For liquid chromatography mass spectrometry (LC-MS) a fritless nanospray column (100 μm ID, packed to 10 cm with 3 μm C18 material (in-house construction) and an LTQ ion trap instrument (Thermo Finnigan; San Jose, CA, USA) equipped with a nanospray source and an UltiMate 3000 HPLC pump (Dionex, Germering, Germany) were used. ESI-MS: m/z [M + H]^+^ = 1012.6 [C_48_H_69_N_13_O_9_S; exact mass: 1011 (calculated)].

### 2.4 [Fe]Fusarinine-C

*Aspergillus fumigatus* strain *∆sidG* was cultured for 36 h at 37 °C at 200 rpm in *Aspergillus* minimal medium containing 1% (m/v) glucose as carbon source, glutamine (20 mM) as nitrogen source, salts, and trace elements, as described previously by Schrettl et al. [15]. Briefly, for preparation of iron-deficient media, iron addition was omitted. Iron-deficient conditions were verified by detection of extracellular siderophore production, which is suppressed by iron. Secreted Fusarinine-C (FSC) was then saturated with iron and purified from the culture supernatant using Amberlite™ XAD18 beads (Dow Chemical Company, Philadelphia, PA, USA) as column matrix. Elution of [Fe]FSC from the Amberlite XAD column was carried out using methanol. [Fe]FSC was then additionally purified via RP-HPLC (gradient F): t_R_ = 8.3 min. MALDI TOF-MS: m/z [M + H]^+^ = 780.4, [M + Na]^+^ = 802.4 [C_33_H_51_FeN_6_O_12_; exact mass: 779 (calculated)].

### 2.5 Conjugation of [Fe]FSC with *N*-Acetyl-L-proline

To [Fe]FSC (1.2 mg, 1.5 μmol) dissolved in 1 mL DMF, *N*-acetyl-L-proline (2.4 mg, 15.4 μmol) in 50 μL was added. After *in situ* activation using HATU/HOAt and adjusting the pH to 9 via DIPEA the solution was allowed to react at RT for 10 h. Hereon, [Fe]FSC-(*N*-acetyl-Pro)_3_ ([Fe]FSC-(AcPro)_3_) was precipitated using diethyl ether, washed several times with diethyl ether and finally dried *in vacuo*. RP-HPLC (gradient A): t_R_ = 12.2 min. MALDI TOF-MS: m/z [M + H]^+^ = 1197.5 [C_54_H_78_FeN_9_O_18_; exact mass: 1196 (calculated)].

### 2.6 FSC-(AcPro)_3_

[Fe]FSC-(AcPro)_3_ (0.9 mg) was dissolved in 2 mL of a solution of disodium ethylenediaminetetraacetic acid (Na_2_EDTA) in H_2_O (25 mM, pH 4). After 12 h reaction time the solution was diluted to 10 ml with H_2_O; FSC-(AcPro)_3_ was purified via a C18 sep-pak cartridge fixation and eluted with CH_3_CN/0.1% TFA. Finally the eluate was evaporated to dryness and the residue dissolved in H_2_O (2.5:1 m/v).

### 2.7 [^68^Ga]FSC-(AcPro)_3_

Labeling was carried out using the fractionated elution method. To 50 μg of FSC-(AcPro)_3_ in 20 μL H_2_O, 100 μL of activity (approx. 35–50 MBq ^68^GaCl_3_ in 0.1 M HCl), and 30 μL sodium acetate solution (1.1 M) were added. The labeling mixture was allowed to react for 15 min at 70 °C and was used without further purification. RP-HPLC: t_R_ = 12.2 min (gradient A).

### 2.8 Conjugation of [Fe]FSC and cyclo(-Arg(Pbf)-Gly-Asp(*O*tBu)-dPhe-Lys(Succ)-)

[Fe]FSC (3 mg, 3.9 μmol) and an excess of 5 eq cyclo(-Arg(Pbf)-Gly-Asp(*O*tBu)-dPhe-Lys(Succ)-) (19.5 mg, 19.2 μmol) were dissolved in 800 μL DMF. After addition of 7.3 mg HATU (19.2 μmol), 2.6 mg HOAt (19.2 μmol) and adjustment of the pH to 9 using DIPEA the reaction mixture was stirred for 72 h at RT. Hereafter, the volume of solvent was reduced *in vacuo*, and the conjugate was precipitated using water. Finally, the precipitate was washed with water and dried to give [Fe]FSC-(cyclo(-Arg(Pbf)-Gly-Asp(*O*tBu)-dPhe-Lys(Succ)-)_3_ (11.2 mg) as a red colored solid. The crude product was used for the next synthesis step without further purification. RP-HPLC (gradient B): t_R_ = 14.0 min.

### 2.9 Deprotection of [Fe]FSC-(cyclo(-Arg(Pbf)-Gly-Asp(*O*tBu)-dPhe-Lys(Succ)-)_3_

For deprotecting the conjugate [Fe]FSC-(cyclo(-Arg(Pbf)-Gly-Asp(*O*tBu)-dPhe-Lys(Succ)-)_3_ (6.4 mg) was dissolved in 1.3 mL of a solution composed of TFA/H_2_O/triisopropylsilan in a ratio of 38:1:1. The reaction mixture was allowed to react for 10 h at RT. Subsequently the solvent was reduced, and crude [Fe]FSC-(RGD)_3_ was obtained by precipitation using diethyl ether. Finally the product was dried and purified via preparative RP-HPLC (gradient G): t_R_ = 15.2 min, yield: 4.7 mg [Fe]FSC-(RGD)_3_.(CF_3_COOH)_3_ (1.5 μmol). RP-HPLC (gradient C): t_R_ = 10.0 min. MALDI TOF-MS [M + H]^+^ = 2836.1 [C_126_H_180_FeN_33_O_39_; exact mass: 2835 (calculated)].

### 2.10 FSC-(RGD)_3_

Demetalation of the [Fe]FSC-(RGD)_3_ was accomplished by dissolving 1.9 mg (0.6 μmol) of the conjugate in 1 mL of a disodium ethylenediaminetetraacetic acid (Na_2_EDTA) solution (25 mM). After 10 h stirring at RT the solution was concentrated *in vacuo*, and FSC-(RGD)_3_ was directly isolated via preparative RP-HPLC (gradient G): t_R_ = 15.9 min, yield: 0.7 mg FSC-(RGD)_3_ (CF_3_COOH)_3_ (0.2 μmol, 37% of theoretical yield). RP-HPLC (gradient C): t_R_ = 10.1 min. MALDI-TOF-MS [M + H]^+^ = 2785.0 [C_126_H_183_N_33_O_39_; exact mass: 2782.3 (calculated)].

### 2.11 [^68^Ga]FSC-(RGD)_3_

Labeling procedure of FSC-(RGD)_3_ was optimized regarding the amount of peptide, type of buffer, and reaction time. Details can be found in Table 1. Overall to 0.5–30 μg FSC-(RGD)_3_ 30 μL sodium acetate solution (1.1 M) and 100 μL ^68^GaCl_3_ of the main fraction of the generator eluate (approx. 35–50 MBq in HCl (0.1 M)) were added. Analysis was performed using RP-HPLC (gradient D): t_R_ = 6.6 min.

**Table 1:** Optimization of the labeling conditions for FSC-(RGD)_3_.

Peptide amount [μg]	Peptide amount [nmol]	A [μL]	B [μL]	pH	Reaction time [min]	RCY [%]	Specific activity [TBq/mmol]
0.5	0.18	30		4.5–5	15	96.0 ± 1.1	214 ± 2.6
2	0.72	30		4.5–5	5	94.5 ± 2.6	53 ± 1.5
2	0.72	30		4.5–5	15	97.2 ± 1.5	54 ± 0.9
2	0.72		20	5	5	95.6 ± 1.7	53 ± 0.9
2	0.72		20	5	15	96.9 ± 0.4	54 ± 0.2
5	1.80	20		3–4	15	94.1 ± 1.5	21 ± 0.7
5	1.80	30		4.5–5	15	98.5 ± 0.7	22 ± 0.1
10	3.59	30		4.5–5	15	99.3 ± 1.3	11 ± 0.1
30	10.78	30		4.5–5	15	99.5 ± 0.8	4 ± 0.03

Optimization reaction time, peptide amount, and buffer systems were modified.

Activity: 100 μL of ^68^GaCl_3_ in 0.1 M HCl (30–50 MBq), reaction at room temperature.

The radiochemical purity was determined by radio-HPLC.

A: 310 mg sodium acetate trihydrate (NaOAc · 3H_2_O) in 2 mL H_2_O (1.1 M, pH 8.3).

B: NaHEPES (1 M)/HEPES (1 M) (2:1) (1.0 M) (1.0 M, pH 8.2).

### 2.12 Distribution coefficient (logD)

To [^68^Ga]FSC-(RGD)_3_ (approx. 0.4 MBq in 50 μL, 125 pmol peptide) diluted in 450 μL phosphor buffered saline (PBS) octanol (500 μL) was added. Hereafter, the mixture was vortexed for 15 min at 1400 rpm and centrifuged for 2 min at 2000 rcf. Subsequently, aliquots of the aqueous and the octanol layer were collected, measured in the gamma counter, and logD values were calculated (n = 5).

### 2.13 Stability assay

Determination of stability of [^68^Ga]FSC-(AcPro)_3_ and [^68^Ga]FSC-(RGD)_3_ was carried out incubating the radiotracers for 30, 60, and 120 min at 37 °C, respectively. Therefore, [^68^Ga]FSC-(AcPro)_3_ or [^68^Ga]FSC-(RGD)_3_ was incubated in 2 mL of PBS (approx. 1.5 MBq, 0.4 nmol), FeCl_3_- (> 1000-fold molar excess of iron), DTPA solution (pH 7.1; > 1000-fold molar excess of DTPA) and in 2 mL (approx. 5 MBq, 1.25 nmol) of fresh human serum, respectively. At selected time points aliquots of PBS-, FeCl_3_-, or DTPA solution were analyzed directly via RP-HPLC, while serum aliquots were mixed with 500 μL CH_3_CN, vortexed, and centrifuged at 20000 rcf for 2 min before analysis. RP-HPLC analysis applying gradient A was used. Extraction efficacy was determined by dividing the activity of the supernatant by the total activity used in this assay.

### 2.14 Protein binding assay

Protein binding ability for [^68^Ga]FSC-(RGD)_3_ was evaluated incubating the radiotracer for 30, 60, and 120 min at 37 °C in fresh human serum. Subsequently, the solutions were passed through a size exclusion spin column (MicroSpin™ G-50 columns, GE healthcare, Buckinghamshire, UK) via centrifugation at 2000 rcf for 2 min. Protein binding ability was determined by measuring the activity bound to the column (non-protein bound) and the activity in the eluate (protein bound) in a gamma counter.

### 2.15 Binding affinity for immobilized α_v_β_3_ integrin (IC_50_ value)

*In vitro* binding affinities of FSC-(RGD)_3_ and cyclo(-Arg-Gly-Asp-dTyr-Val-) (c(RGDyV)) as control were determined by using isolated α_v_β_3_ integrin (Millipore-Chemicon, Temecula, CA, USA) and ^125^I labeled c(RGDyV) as radioligand. The detailed procedure can be found in [16]. Briefly, 96-well plates (Nunc, Thermo Fisher Scientific, Vienna, Austria) were coated for 16 hours at 4 °C with the α_v_β_3_ integrin. Subsequently, the immobilized receptors were incubated with [^125^I]c(RGDyV) and increasing concentrations of the corresponding peptide ranging from 0.001 to 100 nM. The unbound fraction of radioligand was washed out, and receptor bound activity was obtained treating the wells with sodium hydroxide (NaOH) solution (2 M). IC_50_ values were determined by fitting the percent inhibition using OriginPro 8.5 software (Northhampton, MA, USA). Three independent measurements were made.

### 2.16 Internalization assay

M21 (α_v_β_3_ positive) and M21-L cells (negative control) were diluted with RPMI 1640 (Gibco, Invitrogen Corporation, Paisley, UK) containing 1% glutamine (m/v), 1% bovine serum albumin (BSA) (m/v), CaCl_2_ (1 mM), MgCl_2_(1 mM), and MnCl_2_ (10 mM) to a concentration of 2 × 10^6^ cells/mL, and aliquots of 1 mL were transferred to Eppendorf tubes for incubation for 1 h at 37 °C. After addition of [^68^Ga]FSC-(RGD)_3_ (approx. 1.5 × 10^6^ cpm, 22 nM), the cells were incubated in triplicates with either PBS with 0.5% BSA (150 μL, total series) or with 10 μM c(RGDyV) in PBS/0.5% BSA (150 μL, nonspecific series) at 37 °C for 90 min. The incubation was stopped via centrifugation, the medium removed, and the cells were rinsed twice with ice-cold TRIS-buffered saline. Subsequently, the cells were incubated in acid wash buffer (20 mM acetate buffer, pH 4.5) at 37 °C for 15 min. After centrifugation the cells were washed with acid wash buffer, subsequently lyzed by addition of 2 M NaOH, and the radioactivity associated with cells was collected (internalized radioligand fraction). Protein content in the NaOH fraction was determined by way of spectrophotometry using Bradford reagent (Sigma, Vienna, Austria). Internalized activity was determined and expressed as percentage of total activity per milligram protein.

### 2.17 Biodistribution

Biodistribution studies were conducted in compliance with the Austrian animal protection laws and with approval of the Austrian Ministry of Science (BMWF-66.011/0135-II/10b/2008). For the induction of tumor xenografts, M21 and M21-L cells were subcutaneously injected at a concentration of 5 × 10^6^ cells/mouse and were allowed to grow until tumors reached a volume of 0.3–0.6 cm^3^. To determine the α_v_β_3_ receptor specific uptake Balb/c nu/nu mice (Charles River, Sulzfeld, Germany) bearing the human melanoma tumor M21 in the right flank and α_v_β_3_ negative M21-L (as a negative control) in the left flank were used (n = 4). [^68^Ga]FSC-(RGD)_3_ (~ 1 MBq/animal, ~ 0.4 μg peptide, 0.14 nmol) was intravenously injected in the tail vein. The animals were sacrificed by cervical dislocation 60 min post injection, respectively. Organs (heart, stomach, spleen, liver, pancreas, kidneys, and intestine), blood, muscle tissue and tumors were removed and weighted. Activity uptake of the samples was measured in the gamma counter. Results were expressed as percentage of injected dose per gram tissue (% ID/g).

## 3 Results

### 3.1 Peptide synthesis and coupling to the chelator

*N*-acetyl-L-proline was coupled straightforward via its carboxylic function to the amino groups of [Fe]FSC in high yields. After demetalation, FSC-(AcPro)_3_ was purified via sep-pak cartridge for preliminary proof of radiolabeling.

Cyclo(-Arg(Pbf)-Gly-Asp(OtBu)-dPhe-Lys(Succ)-) could be synthesized in good yield. Assembly of the linear RGD peptide was accomplished on solid phase using Fmoc-protocols. Cyclization was carried out under high dilution conditions and subsequent partial Z-deprotection under hydrogen atmosphere. In a next step the lysine side chain was modified via addition of succinic acid enabling the coupling of the RGD peptide to the primary amino functions of [Fe]FSC. Amidation of cyclo(-Arg(Pbf)-Gly-Asp(OtBu)-dPhe-Lys(Succ)-) with [Fe]FSC was accomplished via *in situ* activation using HATU/HOAt and DIPEA (a representative HPLC chromatogram is found in supplementary material Fig. S1). Complete deprotection and subsequent demetalation of the organometalic complex resulted in an average yield of approx. 40% FSC-(RGD)_3_. After HPLC purification the chemical purity (CP) of FSC-(RGD)_3_ amounted to > 95% (see supplementary material Fig. S2).

### 3.2 Radiolabeling

[^68^Ga]FSC-(AcPro)_3_ and [^68^Ga]FSC-(RGD)_3_ were obtained in radiochemical yields (RCY) > 94% comparable to ^68^Ga-labeling of NODAGA-RGD. Standard conditions of 30 μg of FSC-(RGD)_3_ in 30 μL H_2_O, 100 μL of ^68^GaCl_3_ in 0.1 M HCl and 30 μL sodium acetate solution (1.1 M) reacting for 15 min were applied for *in vitro* and *in vivo* tests and the radiolabeling solution used without further purification.

For optimization of the radiolabeling process of FSC-(RGD)_3_ different amounts of peptide (0.5–30 μg), varying time points (5–30 min), different buffer systems (NaOAc or NaHEPES/HEPES) and buffer amounts (15–60 μL), as well as different reaction temperatures (RT:70 °C) were evaluated. Specific activities (SA) ranged from 4 to 200 TBq/mmol. A selected list of radiolabeling data is presented in Table 1 showing that 0.5 μg (0.18 nmol) of FSC-(RGD)_3_ was sufficient to be labeled with ^68^Ga even at RT without further purification with an RCP greater than 95% achieving an SA of 200TBq/mmol. Whereas under the conditions applied a pH of 4.5–5 resulted in a somewhat higher RCP as compared to pH 3-4, no significant differences were found in the buffer type used (HEPES vs. acetate) or when comparing incubation at RT vs. 70 °C.

### 3.3 *In vitro* characterization

Distribution coefficient (logD) of [^68^Ga]FSC-(RGD)_3_ was − 3.6 revealing a hydrophilic character.

Serum protein-bound activity of [^68^Ga]FSC-(RGD)_3_ was approx. 10% of total activity after 30 min incubation in serum, and this value remained stable for the whole monitoring period.

**Fig. 2:**
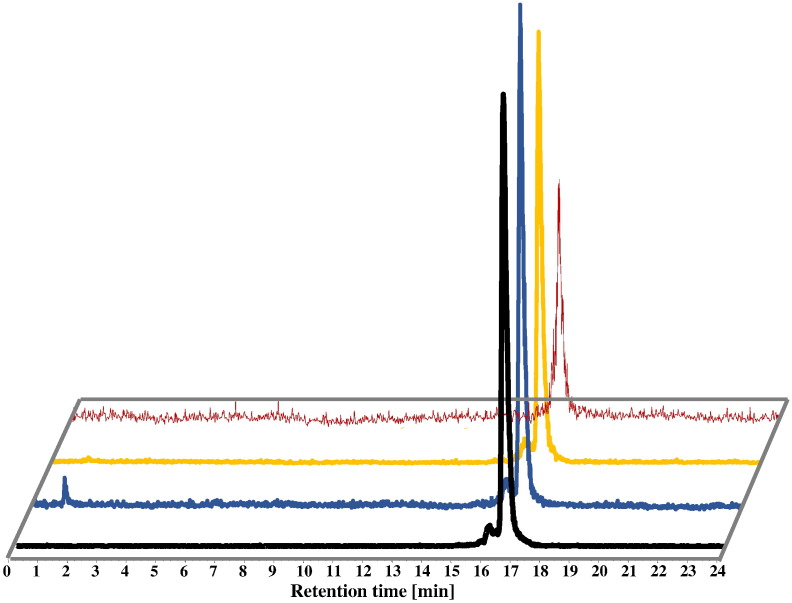
HPLC radiochromatograms of [^68^Ga]FSC-(RGD)_3_: (front/black: radiolabeling solution, middle: stability after 120 min incubation at 37 °C in DTPA solution (blue), FeCl_3_ solution (yellow) and (last) fresh human serum (red).

Both [^68^Ga]FSC-(AcPro)_3_ and [^68^Ga]FSC-(RGD)_3_ were stable in PBS, FeCl_3_, and in DTPA solution as well as in fresh human serum at 37 °C for 2 h, respectively (Fig. 2). Extraction efficiency of [^68^Ga]FSC-(RGD)_3_ in serum was approx. 85%. Protein binding and stability data are summarized in Table 2.

**Table 2:** Protein binding and stability values after 30, 60, and 120 min incubation at 37 °C.

Incubation time [min]	A [%]	B [%]	C [%]	D [%]
30	10.6	99.9	99.3	98.1
60	9.55	99.9	99.1	98.1
120	10.0	99.9	99.2	97.0

A: serum protein binding.

B: stability in fresh human serum.

C: stability in FeCl_3_ solution.

D: stability DTPA solution.

Increasing amounts of FSC-(RGD)_3_ or c(RGDyV) as control successfully suppressed the binding of [^125^I]c(RGDyV) to the immobilized α_v_β_3_ integrin. IC_50_ values were 1.8 ± 0.6 nM for FSC-(RGD)_3_ and 3.2 ± 1.2 nM for c(RGDyV).

**Fig. 3:**
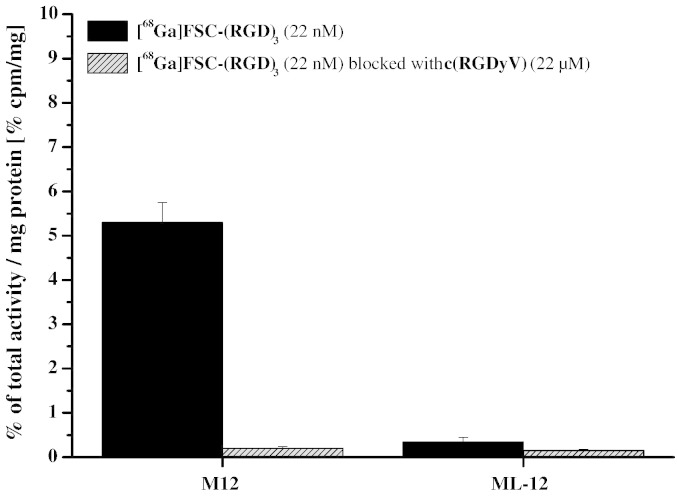
Cell uptake studies using human melanoma M21 (α_v_β_3_ positive) and M21-L (α_v_β_3_ negative) cells showed a receptor specific internalization for [^68^Ga]FSC-(RGD)_3_ (22 nM); binding to receptor negative M21-L and after blocking using c(RGDyV) (22 μM) are negligible.

By incubating [^68^Ga]FSC-(RGD)_3_ (22 nmol) with M21 and M21-L tumor cells and carrying out corresponding blocking studies (22 μmol c(RGDyV)) internalization ability of the multimeric RGD peptide was determined (Fig. 3). The internalized activity was 5.3 ± 0.5% of total activity per milligram protein [% cpm/mg] for α_v_β_3_ positive M21 cells which was reduced to approx. 1/25 of the reference activity via addition of c(RGDyV). In contrast, blocking of M21-L cells just caused a negligible small reduction of accumulated activity decreasing the uptake from 0.3 to 0.2% cpm/mg. Furthermore, the remaining internalized activity in the blocked M21 cells equaled the activity accumulation found for M21-L cells.

### 3.4 *In vivo* characterization

**Fig. 4:**
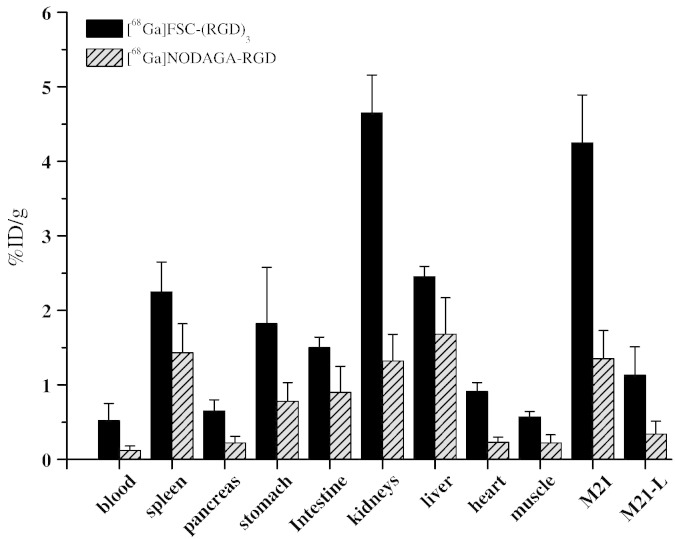
Comparison of biodistribution data of [^68^Ga]FSC-(RGD)_3_ with [^68^Ga]NODAGA-RGD 60 min p.i. (from [12]). For the biodistribution study nude mice bearing the α_v_β_3_-positive human melanoma M21 on the right flank and the negative control tumor M21-L on the left flank were used.

Biodistribution and specific tumor uptake of [^68^Ga]FSC-(RGD)_3_ were determined 60 min postinjection in Balb/c nu/nu mice bearing M21 (α_v_β_3_ positive) as well as M21-L (α_v_β_3_ negative) tumors (n = 4). Collected data are shown in Fig. 4.

Sixty min postinjection the absolute uptake of [^68^Ga]FSC-(RGD)_3_ in the α_v_β_3_ positive M21 tumor was 4.25 ± 0.64% ID/g, but only 1.13 ± 0.38% ID/g in the contralateral negative M21L tumor, being highly significant and confirming the receptor-selective uptake found *in vitro*. Activity concentrations in the different organs ranged from 0.6 ± 0.1%ID/g found for muscle tissue to 4.7 ± 0.5%ID/g for kidneys. Apart from that, activity accumulation in the blood was 0.5 ± 0.2% ID/g resulting in a tumor to blood ratio of 8.2 and a tumor to muscle ratio of 7.4 (in comparison [^68^Ga]NODAGA-RGD: 11.3 and 6.1, respectively).

## 4 Discussion

FSC as starting material for conjugation of targeting sequences was obtained from fungal cultures. Siderophores are produced in large quantities by fungi under iron starvation conditions amounting to up to 10% of their biomass [17]. This provides easy and straight forward access of desferrifusigen and, after addition of Fe(III), of FSC for chemical modifications. E.g., isolation and HPLC purification from a 200 ml culture resulted in more than 20 mg of FSC in high purity.

Derivatization of FSC's amine functions required a selective protection strategy for the hydroxamate groups. Initial attempts to react desferrifusigen, the desferric form of FSC, with HATU/HOAt in DMF and *N*-acetyl-Pro-OH failed in regioselective coupling of *N*-acetyl-Pro-OH to the amino groups of desferrifusigen. Therefore the iron containing peptide siderophore was used as starting material utilizing the iron chelation as protection for the hydroxamate function in analogy to Verel et al. [18]. They used a temporary blocking of hydroxamate groups via addition of Fe^3 +^ for derivatizing Desferrioxamine B (DFO), a linear peptide siderophore, for subsequent antibody conjugation and ^89^Zr-labeling. Applying the same approach starting from FSC initially to synthesize the model compound [Fe]FSC-(AcPro)_3_ and then correspondingly FSC-(RGD)_3_ resulted in a high chemical purity as well as in satisfying overall chemical yields. The straightforward removal of iron from [Fe]FSC-(AcPro)_3_ as well as from [Fe]FSC-(RGD)_3_ in the final step using EDTA at pH 4 can be explained by the phenomenon that hydroxamate based siderophores show a considerably facilitated release of iron under acidic conditions [19]. In contrast to that, at neutral pH both ^68^Ga-labeled FSC conjugates showed almost no sign of transchelation in DTPA solution. Moreover, both compounds were very stable in serum and all challenging solutions during the *in vitro* testing which was comparable with [^68^Ga]NODAGA-RGD, indicating high stability for *in vivo* targeting [12].

Radiolabeling of FSC-(RGD)_3_ using standard protocols also applied to [^68^Ga]NODAGA-RGD resulted in a high RCP at RT within minutes at high specific activities with little influence of reaction conditions such as temperature or buffer type. Without pre-concentration or purification of the ^68^Ga eluate specific activity of up to 200 TBq/mmol was reached. Recently, Notni et al. [20] introduced another trimeric RGD peptide, ^68^Ga-TRAP-(RGD)_3_, based on a (1,4,7-triazacyclononane-1,4,7-tris[methyl(2-carboxyethyl)phosphinic acid]) (TRAP) structure. This TRAP chelating system forms highly stable complexes with Ga^3 +^ allowing efficient radiolabeling over a wide pH range and a straightforward conjugation of three targeting-vectors via amidation without affecting the integrity of the complexation site, concordant with the concept presented here for FSC conjugation. They report that specific activities in the range of 5000 TBq/mmol using 1 GBq of ^68^Ga are routinely achievable [21].

It can be expected for FSC based constructs that considerably higher specific activities are achievable by further optimization of the radiolabeling procedure and appropriate eluate post processing methods [22], [23]. Further studies are currently ongoing using a new, high activity generator as well as pre-concentration to fully test the specific activity limit of FSC-based bioconjugates.

Distribution coefficient (logD) of [^68^Ga]FSC-(RGD)_3_ was − 3.6 which is the same as for the monomeric [^68^Ga]NODAGA-RGD revealing a very hydrophilic character. In contrast to that, protein binding was 10%, which remained stable for the whole monitoring period, as compared to five-fold lower values for [^68^Ga]NODAGA-RGD.

A high binding affinity to the isolated α_v_β_3_ integrin for [^68^Ga]FSC-(RGD)_3_ was found. Its IC_50_ value was in the low nanomolar range, significantly lower as compared to [^68^Ga]NODAGA-RGD (1.8 vs. 4.7 nM, respectively). This also explains the considerably improved binding to α_v_β_3_ integrin expressing cells. An approx. 16-fold higher activity found in the α_v_β_3_-positive cells than in the negative control proved a receptor-mediated mechanism. A remarkable enhancement of internalized activity in comparison to [^68^Ga]NODAGA-RGD or other monomeric RGD derivatives [3], [16] was observed. Moreover, only α_v_β_3_-positive cells could be blocked via addition of c(RGDyV) leading to 1/25 of total activity in comparison to M21 cells additionally confirming the receptor-mediated binding and uptake.

In addition to *in vitro* tests, receptor-specificity was confirmed in a biodistribution study using nude mice bearing M21- and M21-L tumors showing a four-fold higher uptake in the M21- than in the receptor negative M21-L tumor 60 min after injection. In this study not the highest achievable specific activity of [^68^Ga]FSC-(RGD)_3_ was used in order to provide comparable conditions with previous data of radiolabeled RGD peptides in the same tumor model using the same mouse strain, gender and weight. This resulted in a more than threefold higher *in vivo* tumor accumulation as compared to [^68^Ga]NODAGA-RGD (4.3% vs. 1.3% ID/g). We believe that higher specific activities will not yield better targeting properties at least in this model, as it has been shown that even co-injection of 6 mg/kg c(RGDfV) still allowed receptor specific visualization of M21 [24].

The development of multivalent radiopharmaceuticals presenting more than one targeting vector has recently attracted considerable interest. It is assumed that these multimeric compounds on the one hand improve the receptor affinity due to the avidity effect and on the other hand prolong the retention in the target due to increased apparent tracer concentration which appears to have advantages for *in vivo* imaging [25], [26], [27]. Especially larger molecules containing more than one binding epitope may lead to simultaneous multivalent binding [28], [29]. However, the larger size of these constructs also increased uptake in other organs (kidneys, muscle, control tumor, etc) that negatively impact contrast was found. A comparable behavior was found for [^68^Ga]FSC-(RGD)_3_, with higher tumor uptake in tumor but also higher retention in nontarget tissue. Comparing our trivalent FSC-RGD conjugate with the recently described TRAP-(RGD)_3_ very similar properties were found. ^68^Ga-TRAP-(RGD)_3_ showed a logD value of − 3.9 revealing a high hydrophilicity vs. − 3.6 of [^68^Ga]FSC-(RGD)_3_ and an absolute tumor uptake *in vivo* of 5.3 ± 1.3 versus 4.3 ± 0.6% ID/g for [^68^Ga]FSC-(RGD)_3_) using the same tumor model [20], showing the suitability of FSC based trimeric bioconjugates for preparation of ^68^Ga labeled molecules for efficient targeting. Additionally, using appropriate protection strategies for 1 or 2 amines of FSC also mono- and divalent constructs should be chemically accessible; work towards this is currently ongoing as well as tests to label with other radiometals such as ^89^Zr, furthermore optimization of radiolabeling protocols in particular towards moderate radiolabeling conditions (pH, temperature) and testing the influence of metals on radiolabelling. The presented data proved the concept of using FSC based bioconjugates for ^68^Ga labeling as a promising tool for the radiopharmaceutical scientist to develop targeted radiopharmaceuticals for a variety of applications. Further studies in the evaluation of this novel chelating scaffold are required to identify the true potential of this development.

## 5 Conclusion

These initial data show that FSC can be used as bifunctional chelator for synthesis of trimeric bioconjugates allowing ^68^Ga radiolabeling within minutes at RT with high RCY at high specific activities. *In vitro* tests revealed for [^68^Ga]FSC-(RGD)_3_ a strong hydrophilic character as well as high stability towards all challenging solutions proved our hypothesis. Moreover, [^68^Ga]FSC-(RGD)_3_ shows a high affinity for the α_v_β_3_ integrin in the low nanomolar range as well as an elevated internalized activity for the α_v_β_3_-positive M21 cells, whereas binding to M21-L as well as after blocking is negligible receptor specificity. The biodistribution study confirmed the data found *in vitro* and additionally revealed considerably improved tumor targeting in comparison to [^68^Ga]NODAGA-RGD, supporting the concept of using FSC as basis for development of novel targeted bioconjugates for PET applications.

## Conflict of interest

The authors declare that they have no conflict of interest.

## Appendix A Supplementary data

Fig. S1. HPLC chromatogram of conjugation of [Fe]FSC with cyclo(-Arg(Pbf)-Gly-Asp(OtBu)-dPhe-Lys(Succ)-), 72 h reaction time: peak 1: activated RGD, t_R_ = 8.0 min; peak 2: [Fe]FSC-((Pbf, OtBu)RGD)_3_, t_R_ = 14.0 min; [Fe]FSC elutes at the void volume; gradient B. Fig. S2. top: HPLC chromatogram of FSC-(RGD)_3_ after purification, t_R_ = 10.1 min; gradient C, bottom, corresponding MALDI-TOF-MS showing a mass of 2484.978 [M + H]^+^ [C_126_H_183_N_33_O_39_; exact mass: 2782.3 (calculated)].
