# Supplementary material for: [68Ga]FSC-(RGD)3 a trimeric RGD peptide for imaging αvβ3 integrin expression based on a novel siderophore derived chelating scaffold—synthesis and evaluation
Source: Nucl Med Biol. 2015 Feb;42(2):115–22. doi: 10.1016/j.nucmedbio.2014.10.001 (PMC4289911; doi:10.1016/j.nucmedbio.2014.10.001)
Supplement: Supplementary file 1 — Fig. S1. HPLC chromatogram of conjugation of [Fe]FSC with cyclo(-Arg(Pbf)-Gly-Asp(OtBu)-dPhe-Lys(Succ)-), 72 h reaction time: peak 1: activated RGD, tR = 8.0 min; peak 2: [Fe]FSC-((Pbf, OtBu)RGD)3, tR = 14.0 min; [Fe]FSC elutes at the void volume; gradient B. Fig. S2. top: HPLC chromatogram of FSC-(RGD)3 after purification, tR = 10.1 min; gradient C, bottom, corresponding MALDI-TOF-MS showing a mass of 2484.978 [M + H]+ [C126H183N33O39; exact mass: 2782.3 (calculated)]. [file mmc1.docx]

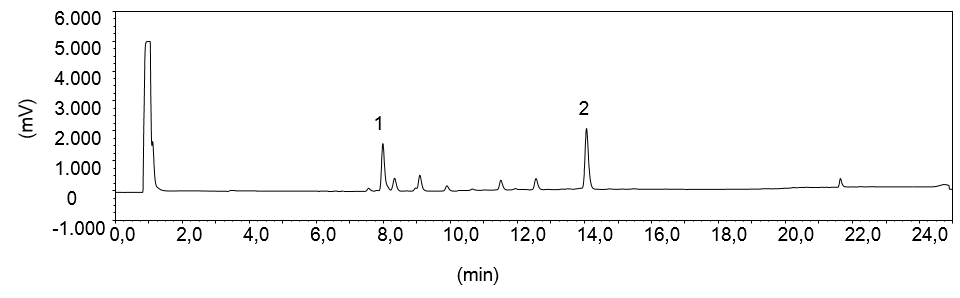


Figure S1 HPLC chromatogram of conjugation of [Fe]FSC with cyclo(-Arg(Pbf)-Gly-Asp(OtBu)-dPhe-Lys(Succ)-), 72 h reaction time: peak 1: activated RGD, t_R_ = 8.0 min; peak 2: [Fe]FSC-((Pbf, OtBu)RGD)_3_, t_R_ = 14.0 min; [Fe]FSC elutes at the void volume; gradient B


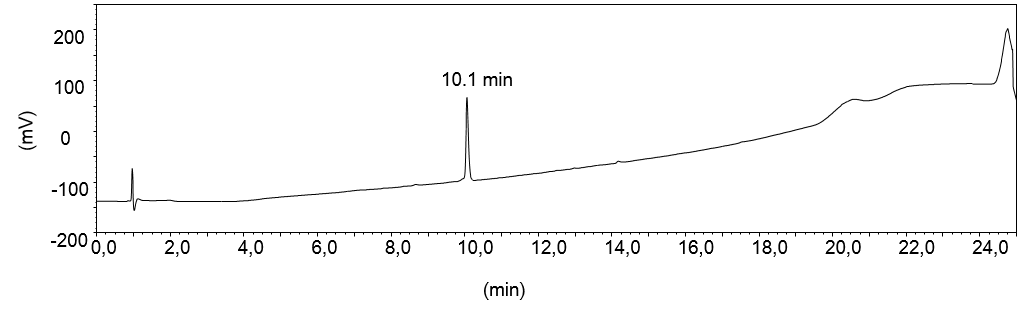

Figure S2 top: HPLC chromatogram of FSC-(RGD)_3_ after purification, t_R_ = 10.1 min; gradient C, bottom, corresponding MALDI-TOF-MS showing a mass of 2484.978 [M + H]^+^ [C_126_H_183_N_33_O_39_; exact mass: 2782.3 (calculated)]
